# Application of FBG Sensing Technology in Stability Analysis of Geogrid-Reinforced Slope

**DOI:** 10.3390/s17030597

**Published:** 2017-03-15

**Authors:** Yijie Sun, Hongzhong Xu, Peng Gu, Wenjie Hu

**Affiliations:** College of Transportation Science & Engineering, Nanjing Tech University, Nanjing 210009, China; sunnju@njtech.edu.cn (Y.S.); gupengkim@163.com (P.G.); stuwjhu@163.com (W.H.)

**Keywords:** FBG, geogrid, slope, numerical modeling, stability analysis

## Abstract

By installing FBG sensors on the geogrids, smart geogrids can both reinforce and monitor the stability for geogrid-reinforced slopes. In this paper, a geogrid-reinforced sand slope model test is conducted in the laboratory and fiber Bragg grating (FBG) sensing technology is used to measure the strain distribution of the geogrid. Based on the model test, the performance of the reinforced soil slope is simulated by finite element software Midas-GTS, and the stability of the reinforced soil slope is analyzed by strength reduction method. The relationship between the geogrid strain and the factor of safety is set up. The results indicate that the measured strain and calculated results agree very well. The geogrid strain measured by FBG sensor can be applied to evaluate the stability of geogrid-reinforced sand slopes.

## 1. Introduction

It is common to improve the stability of a slope using reinforced geosynthetics such as geogrids and geotextiles. This method is simple and convenient for construction with low cost, and has been widely used in practical engineering [1]. Many researchers have conducted a variety of laboratory and field tests on the geogrid reinforcement behavior. It is found that the internal stability of the geogrid-reinforced slope is affected by the direct sliding and pullout resistance along the soil–geogrid interface and the tensile resistance of geogrid layers [2,3,4]. Therefore, accurate deformation/force measurement of the geogrid is important for the design and proper assessment of reinforced slopes [5,6].

Numerous investigations on the measurement of geogrid internal force have been carried out by various methods. Bathurst et al. [1] used a high-elongation strain gauge and found a nonlinear relationship between the local and global strain of the geogrid. Eekelen et al. [7] employed bicycle gear cables to measure strains within the geosynthetic. Yang et al. [8] employed flexible displacement sensors to study the field behavior of a geogrid-reinforced soil retaining wall. However, conventional sensors are usually inconvenient to install in the field and are susceptible to electromagnetic interference (EMI), affecting the accuracy of monitoring results.

In the last few decades, fiber Bragg grating (FBG) technology has developed quickly. The civil engineering community has successfully used FBG sensors for structural health monitoring due to their unique advantages over traditional electrical-based sensors, such as their light weight and small size, immunity to EMI, waterproof property, high sensitivity, and excellent long term durability. Some researchers have employed FBGs to geotechnical-related health monitoring. Pei et al. [9] improved an FBG in-place inclinometer for slope movement and demonstrated its higher sensitivity compared to conventional inclinometers. Zhu et al. [10] used FBGs to monitor the performance of a soil nailed slope and found that the tensile forces of nails had a close relationship with rainfall infiltration and soil mass movement. Zamara et al. [11] successfully applied FBGs to measure the geosynthetic reinforced landfills in the field. The FBG-based smart geogrids can achieve both reinforcement and sensing capabilities.

The limit equilibrium method (LEM) is the main method to investigate geogrid-reinforced slope stability. Blatz and Bathurst [12] conducted three large-scale laboratory model tests and analyzed the bearing capacity of the slope using LEM. However, LEM-based models do not achieve the deformation simulation. The finite element method (FEM)-based numerical approach is often used to simulate the laboratory. Based on FEM, Tanchaisawat et al. [13] investigated the reinforced behavior of geogrids in soft clay, and Li et al. [14] studied the viscous behavior of geogrid-reinforced sand, providing satisfactory prediction with the test results. Recently, a strength reduction method based on FEM was developed. It is useful to get the stability state and strain distribution of a reinforced body, which are helpful for the investigation of the deformation and failure laws of geogrid-reinforced slopes.

In this paper, a static loading test on a medium-sized geogrid-reinforced slope model was conducted in the laboratory. FBG sensors were installed along the geogrid to measure its strain distribution. Using the FEM-based numerical model, the relationship between the characteristic strain of the geogrid and the factor of safety of the model slope was established. The analysis results verify the effectiveness of the FBG sensing technologies in the performance monitoring and stability elevation of a reinforced slope.

## 2. Model Test

**Figure 1:**
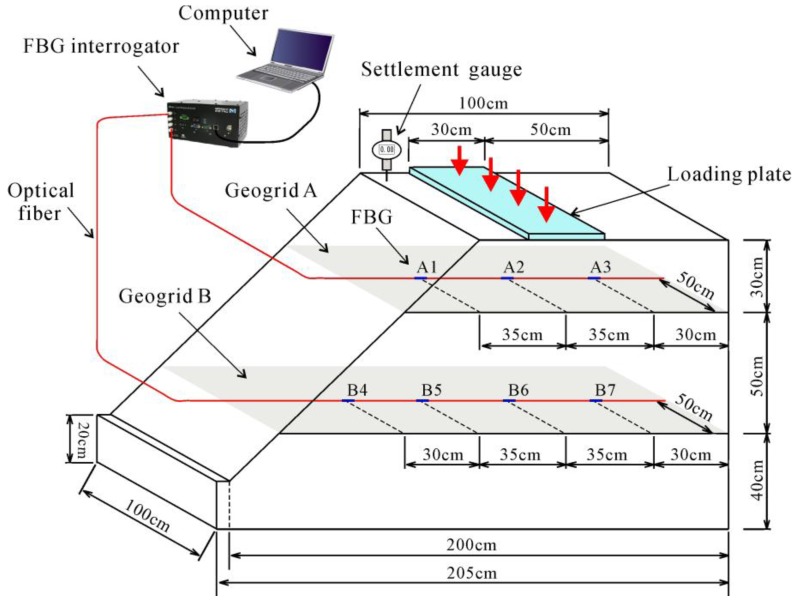
General arrangement of the geogrid-reinforced slope and locations of fiber Bragg grating (FBG) sensors.

**Figure 2:**
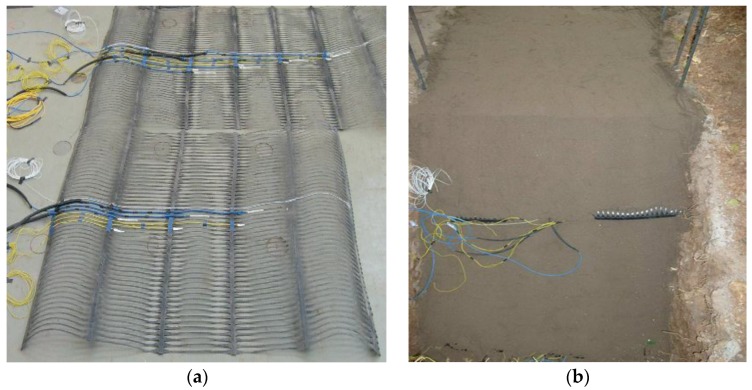
Photographs of the geogrid and **s**lope model: (**a**) geogrid installed with sensors; (**b**) upward view of the slope.

In this paper, a laboratory geogrid-reinforced sand slope model test was conducted. The size of the slope model was 2.05 m long, 1 m wide, and 1.2 m high. The slope inclination was 45°. A hydraulic jack which rested on a thick iron plate of 0.3 m length was used to apply load on the slope crest. It was 20 cm away from the slope shoulder. Figure 1 shows more details about the setup of the model test. Figure 2 shows a photo of the geogrid installed with sensors and the reinforced slope model.

The slope consists of fine sand with two horizontal layers of bi-directional geogrid marked as geogrid A and geogrid B. The arrangement of the measurement point is presented in Figure 1. As illustrated in Figure 1 and Figure 2a, a total of seven FBG sensors were installed along the ribs of the geogrid. Wavelengths of FBGs were measured by interrogator type SM125. It should also be noted that seven electrical resistance strain gauges were installed in addition to the FBGs for contrast. However, only one electrical strain gauge at location A1 survived to completion of the experiment, while none of the FBGs broke down. The properties of the soil used in this test are given in Table 1. The load was applied in a direction perpendicular to the top surface of the model with an increment of 3 kN (i.e., 10 kPa) under manual control.

Each new step load was applied on the model as long as the monitoring data of the strain sensors changed less than 10 με per minute. The parameters of the geogrid are shown in Table 2.

## 3. Numerical Simulation of Slope Model

**Figure 3:**
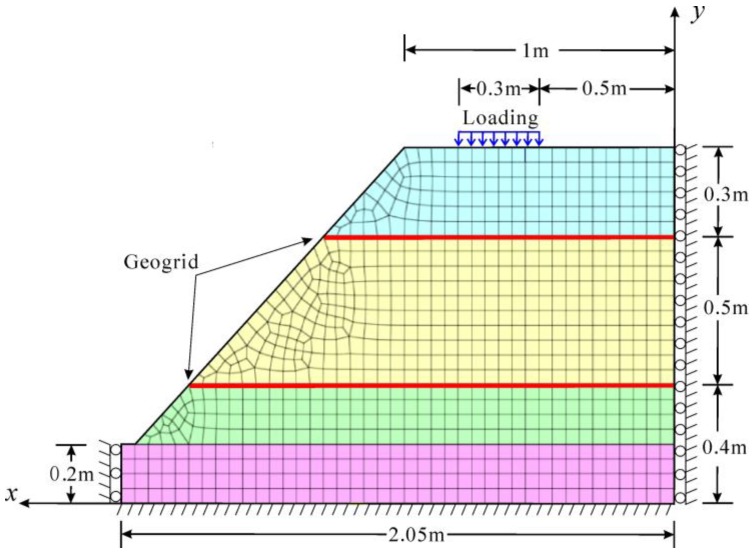
Finite element model.

In this paper, the finite element model is built by the software Midas-GTS, as shown in Figure 3. The geometry size of the numerical model is consistent with that of the physical model. The model has two supporting constraints: the left and right sides were fixed with the displacement in the x direction, while both the x and y directions were fixed for the model’s bottom. Surcharge load increased by 3 kN per step. The parameters of the soil for the finite element analysis are presented in Table 1, which uses the Mohr–Coulomb model. The structural unit (1D) provided by Midas was employed to simulate the geogrid. The weight of geogrid and its ability to bear compressive stress were ignored. Springs connections between the geogrid and the soil were defined.

Figure 3 shows the numerical model of slope, in which the red lines represent two horizontal layers of geogrid, the blue rectangle represents the loading area. The stability of geogrid-reinforced soil slope is analyzed by strength reduction method. The factor of safety of the slope is calculated.

## 4. Test Results and Analysis

### 4.1. Strain Monitoring Results

**Figure 4:**
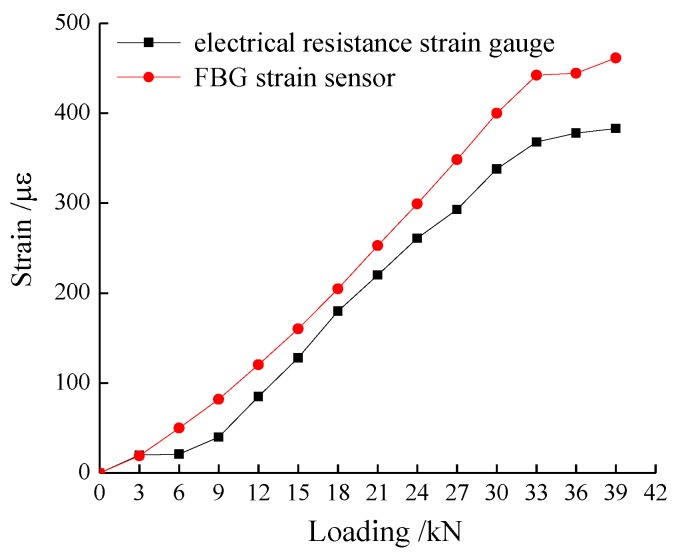
Comparison between FBG and electrical resistance strain gauge test results.

Figure 4 shows the monitoring results of the FBG and the electrical resistance strain gauge at location A1 under different surcharge loads. The data agree well with each other, indicating that the FBG could measure the strain of geogrid accurately. The strain value increment rate of both FBG and electrical resistance strain gauge decreased when the load was larger than 33 kN.

**Figure 5:**
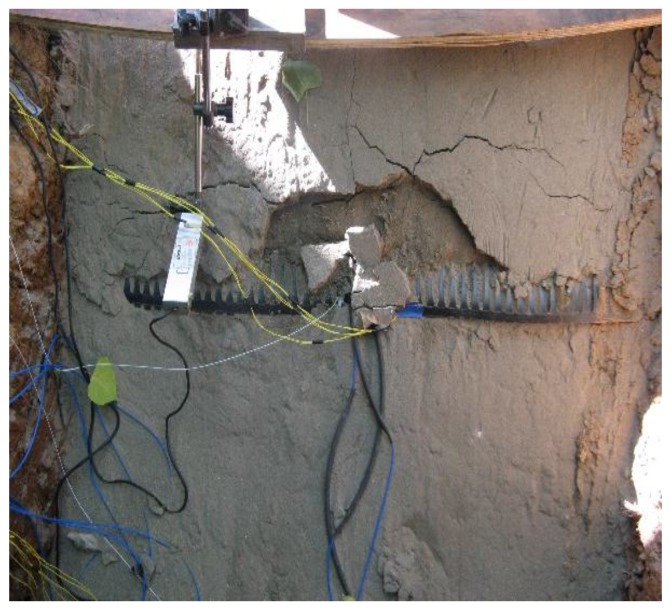
Crack development and damage of slope in the test.

When the load increased from 33 kN to 36 kN, some visible cracks appeared on the slope surface, and these cracks developed gradually. When the load increased to 39 kN, the cracks connected to each other and the slope moved along the failure surface, as shown in Figure 5.

**Figure 6:**
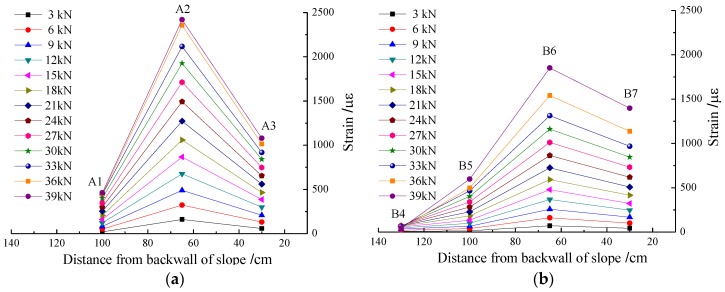
FBG-measured strain distribution of geogrid under different loads. (**a**) Geogrid A; (**b**) Geogrid B.

Figure 6a,b shows the strain distribution of the geogrid under different loads in geogrid A and geogrid B. The results indicate that the FBG strain values of point A2 and B6 under the loading area are the largest in the horizontal direction, and that the strain decreased gradually to both sides. A more sharply decreasing rate was seen on the side close to the front surface of the slope compared to that near the back edge.

### 4.2. Comparison between the Test and the Numerical Simulation

**Figure 7:**
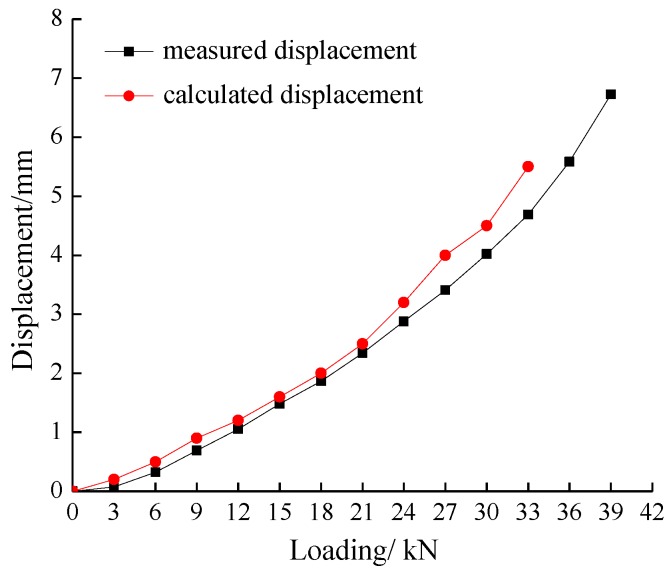
The load–displacement curves for the test and numerical simulation.

Figure 7 shows the vertical load–displacement results in the middle of the loading plate for the test and the numerical simulation. The settlement in the slope crest increases approximately linearly during the initial loading period, and the numerical simulation and monitoring results are in good agreement with each other. However, the deviation increases when approaching critical loads. The numerical simulation results do not converge when the load increases to 36 kN, indicating that a certain scale of slip surface formed and became continuous. However, the settlement gauge increased gradually until 39 kN. The heterogeneity and crack propagation of the soil led to some local failure of the soil mass. This may be one of the main reasons for the deviation between the test and simulation results during the late loading stages.

**Figure 8:**
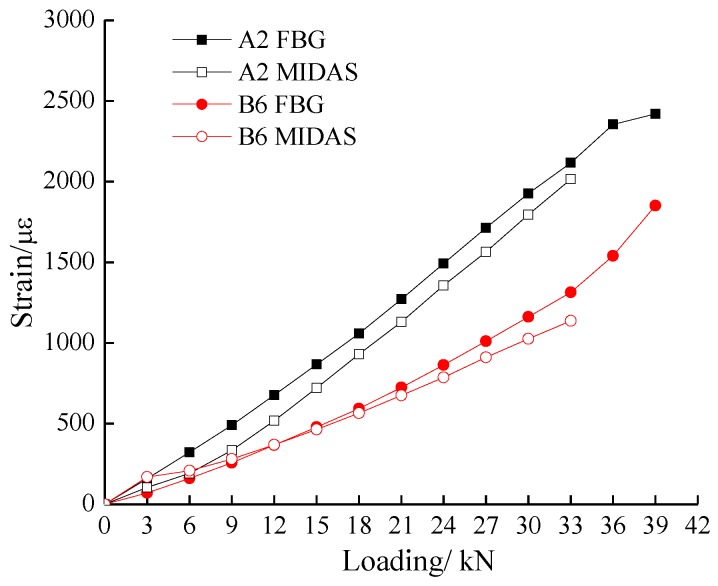
The comparison of test and numerical simulation results.

The comparison of test and numerical simulation results between measurement point A2 and B6 is depicted in Figure 8. At the beginning of the loading, the strain value and the increment rate of the geogrid measured by FBG was larger than that of geogrid B, meaning that the geogrid A played a major role in the reinforcement of the slope. With the increase of load, their values show a trend suggesting that geogrid B begins to perform a better reinforcement effect.

## 5. The Evaluation of Reinforced Sand Slope Stability

According to numerical simulation, it was found that the stress/strain state as well as the stability condition of the geogrid reinforced slope changed under the action of surcharge loads. With the propagating of the plastic zones, strains of the geogrid will accumulate. Additionally, the monitoring results demonstrate that the strain state of geogrid has a good correlation with the load increment. Therefore, there should be a close relationship between the geogrid strain and the factor of safety of the slope.

Zhu et al. [15,16] found that the average maximum horizontal strain of soil mass at different elevation has a good empirical relationship with the factor of safety of the unreinforced fill slope. Analogously, a characteristic geogrid strain is introduced here, which is defined as the average of maximum strains of geogrid; i.e.,

(1) $${\bar{\varepsilon }}_{g}=\frac{\sum_{i=1}^{n}\max(\varepsilon_{gi})}{n}$$

where $\varepsilon_{gi}$ is the measured geogrid strain at the i-th strain monitoring section (i = 1, 2, …, *n*), and ${\bar{\varepsilon }}_{g}$is the average of measured geogrid strain.

Equation (2) is used to describe the empirical relationship between the geogrid strain and the factor of safety.

(2) $$K_{g}=a{\left({\bar{\varepsilon }}_{g}\right)}^{b}$$

where *K_g_* is the factor of safety calculated with strength reduction method and *a* and *b* are two fitting parameters.

**Figure 9:**
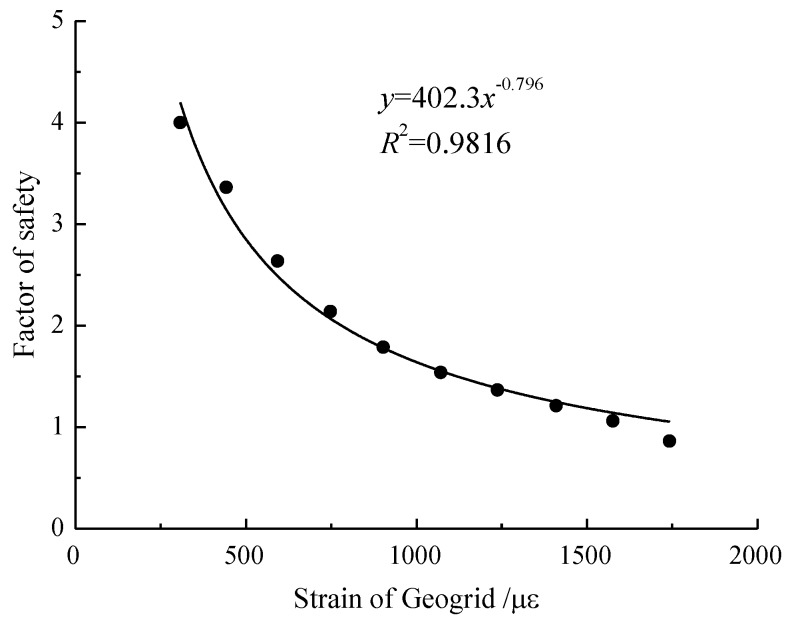
Relationships between the factor of safety and geogrid characteristic strain.

According to the results of the test model in this paper, parameters are fitted as *a* = 402.3, *b* = −0.796. The correlation coefficient *R*^2^ is found to be 0.9816 (Figure 9). As the value of geogrid strain increases, the factor of safety of the slope decreases gradually.

**Figure 10:**
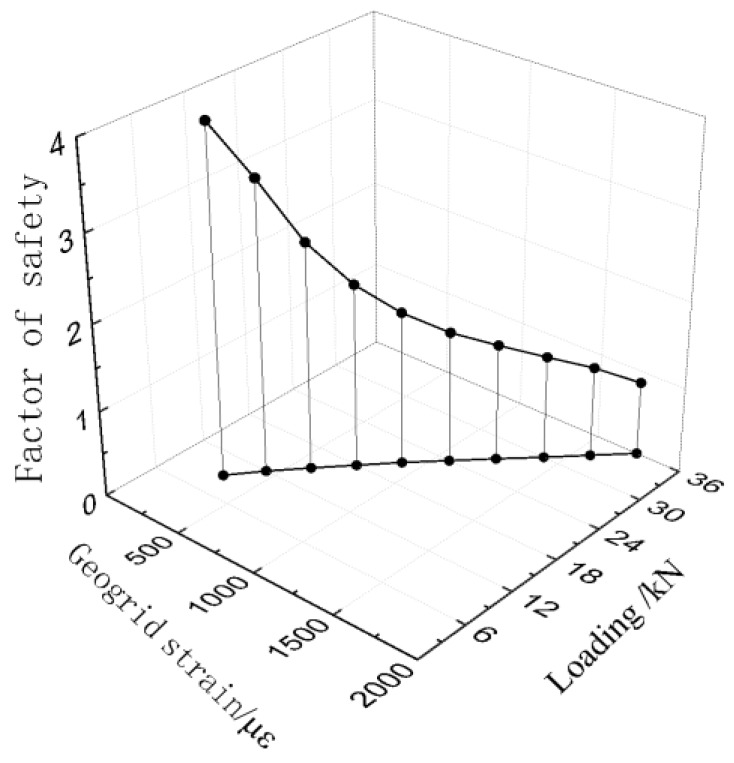
Relationship between loading, characteristic maximum strain, and the factor of safety.

Figure 10 shows the good relationship between the loading, the factor of safety, and the strain of the geogrid. It illustrates that as the loading on the slope crest increases, the factor of safety of the slope decreases as the geogrid strain increases gradually. Therefore, the strain state of geogrid can be used to evaluate the factor of safety of this model slope as well as the surcharge load condition.

## 6. Conclusions

A geogrid-reinforced sand slope model test was conducted. A static loading test was carried out. A quasi-distributed optical fiber sensing system for measuring the longitudinal strain of the geogrid was installed based on FBG sensing technology. The conclusions are summarized as follows:

1. The FBG sensors were employed to measure the strain distribution of the geogrid effectively.
2. There was an empirical relationship between the geogrid strain and the factor of safety for the model slope. This result indicates that the geogrid strain can be applied to evaluate the stability of the slope.

This study shows the potential of FBG-based monitoring technology in the analysis of slope stability.

However, it is worth pointing out that the temperature effect on the FBG monitoring data is overlooked in the present paper because of the short test period. In the long-term monitoring of the actual slope, the temperature influence on the monitoring results should be considered seriously by using different temperature compensation methods according to the field conditions. Another important factor that was neglected is the scale factor effect. Full-scale and field tests are critical to improve the understanding of the behavior of reinforced soil slopes. Therefore, further studies will be conducted to determine the feasibility of applying this technology-based method to evaluate the stability of a reinforced slope in real situations considering these the factors.

## Figures and Tables

**Table 1:** Properties of the soil in the model test.

Bulk Density (kN/m^3^)	Density (kg/m^3^)	Elastic Modulus (MPa)	Poisson’s Ratio	Internal Friction Angle (°)
20	1549	100	0.3	36

**Table 2:** Properties of the geogrid.

Material	Ultimate Longitudinal Tensile Strength (kN/m)	Longitudinal Strain/MPa (%)
Polypropylene	83.5	9.7
